# The influence of population stratification on genetic markers associated with type 1 diabetes

**DOI:** 10.1038/srep43513

**Published:** 2017-03-06

**Authors:** Karla Fabiana Brasil Gomes, Aritânia Sousa Santos, Cintia Semzezem, Márcia Regina Correia, Luciano Abreu Brito, Marcelo Ortega Ruiz, Rosa Tsuneshiro Fukui, Sergio Russo Matioli, Maria Rita Passos-Bueno, Maria Elizabeth Rossi da Silva

**Affiliations:** 1 Laboratório de Carboidratos e Radioimunoensaio (LIM-18) do Hospital das Clínicas da Faculdade de Medicina da Universidade de São Paulo. Av. Dr. Arnaldo, 455, sala 3324, 01246-903, São Paulo, São Paulo, Brazil; 2 Departamento de Genética e Biologia Evolutiva, Instituto de Biociências da Universidade de São Paulo. Rua do Matão, 277, 05422-970, São Paulo, São Paulo, Brazil; 3 Laboratório de Imunologia, Rua Claudio Manoel da Costa, 270, Marília, São Paulo, Brazil

## Abstract

Ethnic admixtures may interfere with the definition of type 1 diabetes (T1D) risk determinants. The role of *HLA, PTPN22, INS-VNTR*, and *CTLA4* in T1D predisposition was analyzed in Brazilian T1D patients (n = 915), with 81.7% self-reporting as white and 789 controls (65.6% white). The results were corrected for population stratification by genotyping 93 ancestry informative markers (AIMs) (BeadXpress platform). Ancestry composition and structural association were characterized using Structure 2.3 and STRAT. Ethnic diversity resulted in T1D determinants that were partially discordant from those reported in Caucasians and Africans. The greatest contributor to T1D was the HLA-DR3/DR4 genotype (OR = 16.5) in 23.9% of the patients, followed by -DR3/DR3 (OR = 8.9) in 8.7%, -DR4/DR4 (OR = 4.7) in 6.0% and -DR3/DR9 (OR = 4.9) in 2.6%. Correction by ancestry also confirmed that the DRB1*09-DQB1*0202 haplotype conferred susceptibility, whereas the DRB1*07-DQB1*0202 and DRB1*11-DQB1*0602 haplotypes were protective, which is similar to reports in African-American patients. By contrast, the DRB1*07-DQB1*0201 haplotype was protective in our population and in Europeans, despite conferring susceptibility to Africans. The DRB1*10-DQB1*0501 haplotype was only protective in the Brazilian population. Predisposition to T1D conferred by *PTPN22* and *INS-VNTR* and protection against T1D conferred by the DRB1*16 allele were confirmed. **C**orrecting for population structure is important to clarify the particular genetic variants that confer susceptibility/protection for T1D in populations with ethnic admixtures.

Type 1 diabetes mellitus (T1D), which results from the autoimmune destruction of pancreatic β (beta) cells, is a polygenic disease that is influenced by both genetic and environmental contributing factors^1^. More than 60 loci are involved in susceptibility to T1D. The Major Histocompatibility Complex (IDDM1 locus) has been identified as a major determinant for genetic susceptibility to autoimmune diabetes, with the Human Leukocyte Antigens (HLA)-DR and -DQ alleles providing 40%–50% of the risk. In several countries, increased susceptibility is also conferred by differences in the variable number of tandem repeats at the 5′-end of the insulin gene (*INS-VNTR*) and polymorphisms in immune response genes, including protein tyrosine phosphatase non-receptor type 22 (*PTPN22)* and cytotoxic T-lymphocyte-associated protein 4 (*CTLA4*)^1^.

T1D is more frequent in white populations, but its incidence varies in Caucasians from different countries and within the same country^2^. In Brazil, the estimated frequency of risk polymorphisms for T1D showed inter-regional differences and were usually lower than those in populations referred to as Caucasians in other countries^3^,^4^,^5^, reinforcing the need to expand genetic predisposition studies in the Brazilian population to clarify causal variants and related ancestry. The Brazilian population was formed by a strong admixture from three different ancestral roots, i.e., Amerindians, Europeans, and Africans, thereby hindering ethnic identification (or genetic ancestry) based predominantly on skin color^6^. Another obstacle is population stratification, which arises from ethnic admixture. Subgroups that are ancestrally distinct tend to have significant representation of different genetic ancestries, which suggests that a combination of over-represented alleles in patients relative to controls may be due to the greater frequency of this allele in the predominant ancestral population in cases. By providing detailed information about ancestry, ancestry informative markers (AIMs) increase the ability to detect both spurious associations between population cases and controls and genetic effects with greater accuracy and precision^7^.

Few studies have evaluated the genetic risks of T1D in Brazil, and none have considered ancestry. This approach, can remove the bias due to population stratification of causal polymorphisms on susceptibility to T1D. This study is the largest report of genetic markers for T1D in Brazil and can help identify T1D risk alleles, differentiating causal variants from those related to ancestry.

## Statistical Analysis

The variable distributions were verified by the Kolmogorov-Smirnov test. Qualitative variables were compared using the chi-square test or the Fisher’s exact test, with Woolf corrections when necessary. Numerical variables with parametric and non-parametric distributions were analyzed using Student’s t and Mann-Whitney tests^8^,^9^,^10^,^11^. The Hardy-Weinberg equilibrium was calculated for all of the genotypes using the chi-square test. Bonferroni correction was applied for multiple tests.

The ancestral composition of the subjects in our sample was inferred using Structure 2.3^12^,^13^. Briefly, this program assumed the existence of K parental populations for the tested mixed population (where K was set equal to 3, based on the known European, African, and Amerindian composition of the Brazilian population) and grouped individuals with admixed proximity to each of the parental populations using Bayesian inference. Thus, the fraction of genomic contribution from European, African, and Amerindian populations was obtained for each individual.

After defining the ancestor-estimated contribution, the single nucleotide polymorphisms (SNPs) contained in the candidate genes were tested by structured association analysis using the Association Test Structured Population (STRAT) program. This program provides a statistical method for performing association mapping in structured populations assuming no stratification or conditioning for individual lines. In short, this approach allows a case-control study with correction for the biases introduced by population stratification in association studies^12^. A p-value ≤ 0.05 was considered statistically significant and was equivalent to a 95% confidence level.

## Results

### Population characteristics

The characteristics of the groups are shown in Table 1. Patients with T1D were younger than the controls (p < 0.0001), with a predominance of females (p < 0.0001) and a lower body mass index (BMI, p < 0.0001). White was the most prevalent self-reported skin color in both groups; however, the diabetes group had a greater frequency of white skin color (81.7% vs. 65.6%, p < 0.0001 and lower frequencies of brown (15.4% vs. 27.8%, p < 0.0001) and black (2.4% vs. 5.9%, p = 0.0003) skin color than did the controls.

The average patient age at diagnosis was 12.3 ± 8.4 years and diabetes duration of 12.4 ± 10.6 years. Fasting glucose and HbA1c levels were higher (p < 0.0001) and C-peptide concentrations were lower (p < 0.0001) in patients than in the control group (Table 1).

The pancreatic autoantibodies titers were higher in patients than in controls and were as follows: GAD65A: 10.8 ± 25.4 × 0.12 ± 0.68 IU/mL; IA-2A: 3.5 ± 9.0 × 0.1 ± 0.23 IU/mL; and ZnT8A: 201.8 ± 382.6 × 3.3 ± 11.3 IU/mL (p < 0.0001). Pancreatic autoantibodies were also more frequent in patients: GAD65A: 47.3% × 1.5%; IA-2A: 42.5% × 1.9%; and ZnT8A: 48.9% × 1.6% (p < 0.0001).

### Ancestral composition

**Figure 1:**
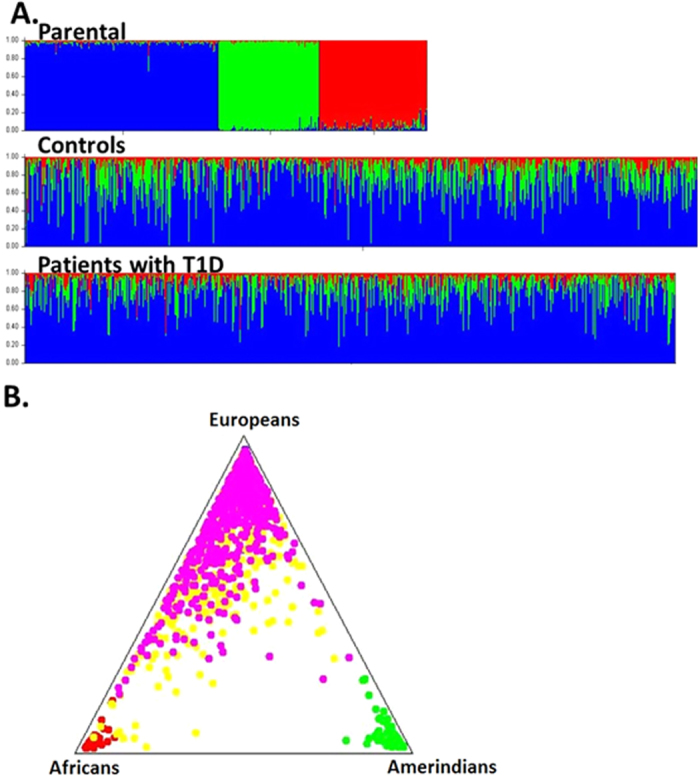
Graphical representations of individual contributions generated by Structure software, assuming k = 3 populations. (**A**) Bar plot showing the African (red), European (blue) and Amerindian (green) contributions. Each column represents an individual, with his grouped populations. The three groups of the first bar-plot represent the parental populations (European, African and Amerindian); (**B**) Tri-plot showing parental populations grouped at the corners and cases and controls around the triangle according to their ancestors. Pink circles represent patients with T1D and yellow circles represent control individuals.

The contribution of each of the three parental populations (European, African, and Amerindian) was obtained for both groups. T1D patients had, on average, 77% European, 15% African, and 7.3% Amerindian ancestries, whereas the healthy controls had, on average, 71% European, 21% African, and 7.9% Amerindian ancestries. The ancestral contributions are shown in Fig. 1.

### HLA genes

The frequencies of the *HLA-DRB1* and -*DQB1* alleles are shown in Tables 2 and 3, respectively, and the -*DRB1/DRB1* genotypes and -*DRB1/DQB1* haplotypes are shown in Tables 4 and 5, respectively. The tables list the individual alleles, genotypes, and haplotypes that were seen at least ten times in the cases and controls. Significance levels of the allele, genotype, and haplotype associations were included, assuming no stratification and after correcting for the effects of stratification by STRAT.

The HLA-DRB1*0301, -*0401, -*0402, -*0405, and -*09 alleles were most prevalent in the T1D group in a decreasing odds ratio hierarchy: -*0405 to *0301, -*0402, -*0401, and -*09. Similarly, protection was associated with the following alleles in an increasing odds ratio hierarchy: HLA-DRB1*0302, -*15, -*10, -*14, -*11, -*13, and -*07. These results were not affected by adjustments for stratification, except allele -*16, which was initially neutral and became protective after correction (p non-structured = 0.0024, p structured < 0.001).

The risk for T1D was also conferred by HLA-DQB1*0201 (OR = 3.52) and DQB1*0302 (OR = 3.33), whereas DQB1*0602, -*0603, -*0503, -*0402 and -*0501 were protective in an increasing odds ratio hierarchy. After correction for stratification only, DQB1*0501 lost its significance as a protective allele (p non-structured = 0.0014, p structured = 0.007).

The genotype that conferred most risk (Table 4) was HLA-DR3/DR4 (OR = 16.5), which occurred in 23.9% of patients, followed by -DR3/DR3 (OR = 8.9) in 8.7%, -DR4/DR4 (OR = 4.7) in 6.0%, and -DR3/DR9 (OR = 4.9) in 2.6%. Protection against T1D was obtained by the absence of the HLA-DR3, -DR4, and -DR9 alleles (OR = 0.1). These results were unaffected by the correction for stratification.

The DRB1*0405-DQB1*0302, -DRB1*0301-DQB1*0201, -DRB1*09-DQB1*0202, -DRB1*0402-DQB1*0302, and -DRB1*0401-DQB1*0302 haplotypes were the most prevalent in the T1D group, whereas -DRB1*11-DQB1*0602, -DRB1*15-DQB1*0602, -DRB1*14-DQB1*0503, -DRB1*13-DQB1*0603, -DRB1*10-DQB1*0501, DRB1*0302-DQB1*0402, -DRB1*11-DQB1*0301, and -DRB1*07-DQB1*0202 conferred protection (Table 5). Neither the susceptibility nor the protection traits of the haplotypes was affected by the correction for stratification using STRAT, except –DRB1*07-DQB1*0201, which was included as a protective haplotype after stratification.

### Associations of *non-HLA genes* variants with T1D

The genotypic frequencies of the INS-VNTR polymorphic alleles CTLA4 (+49 A/G), and PTPN22 1858T are shown in Table 6. The table also shows the association tests with T1D with 95% confidence, assuming no stratification and after correcting for stratification as determined using STRAT.

The CTLA4 genotypes for the controls were not in Hardy-Weinberg equilibrium (p = 0.0001) and were excluded from further analysis, whereas the *PTPN-22* and *INS-VNTR* genotypes were in Hardy-Weinberg equilibrium in both groups. The *INS-VNTR* I/I genotype prevailed in patients with T1D (60.7% × 32.2% of controls) and the PTPN22-1858T: CT + TT pooled genotypes (19.0% of T1D patients × 10.6% of controls), which confer susceptibility to T1D, even after correcting for population stratification.

## Discussion

T1D is a complex autoimmune disease with a strong genetic component^1^,^2^ that is mainly related to HLA genes. HLA genes are the most polymorphic genes in the human genome, which has resulted in a widely variable distribution of HLA alleles and haplotype combinations among populations. Wide variability is also observed in other T1D-predisposing genes, including CTLA4, INS-VNTR, and PTPN22, in several ethnic groups and is likely related to different genetic backgrounds and environmental factors^1^,^2^.

Previous studies conducted in Brazil with small sample sizes have shown inter-regional differences in the frequencies of the HLA-DR and -DQ alleles and of the other risk polymorphisms for T1D^3^. Furthermore, differences in populations referred to as Caucasian in other countries were also observed^14^.

Considering that these discrepancies may result from the heterogeneity of the highly mixed Brazilian population, which may introduce bias into the evaluation of causal disease markers, we expanded our studies to a larger cohort and included a characterization of genetic ancestry to clarify the causal variants and related ancestry. This clarification is important because previous studies^6^ noted a weak correlation between skin color and ancestral genomes in Brazil.

The ancestral composition of our sample was determined using Structure after genotyping 89 AIMs. To determine whether the associations of the alleles, genotypes, and haplotypes with T1D remained even after correcting for the stratification bias in our population, association tests were performed using STRAT, which uses a statistical approach for association mapping candidate genes in structured populations^12^.

Analysis of the 89 AIMs confirmed the three major ancestral roots of the population in our study: European, African, and Amerindian. This result most likely reflects major milestones in our history, specifically the European colonization of Amerindians and African slaves. The highest percentage of European descent in both the T1D group and controls (77% and 71%, respectively) corroborates other reports^6^. The self-reported skin color of the patients in our study reinforces the poor correlation between color and ancestry genomics in Brazil as well as the need for ancestry characterization. The diabetes group had a higher frequency of self-reported whites (81.7% vs. 65.6%, p < 0.0001) and lower frequencies of browns (15.4% vs. 27.8%, p < 0.0001) and blacks (2.4% vs. 5.9%; p = 0.0003) than the controls.

Our results have few, but important, differences from those obtained for Europeans and African Americans. They suggested that the proportion of women among T1D patients (57.6% × 42.4%; p < 0.001) was higher than reported in the literature, which is in accordance with previous reports by our group^5^ and by Gomes MB in a Brazilian multicenter study^15^. However, we cannot exclude some bias in the selection of patients as our study was not designed to estimate the disease prevalence. It is important to remember that there is no difference between genders in most series, except in populations such as those of Sardinia (Italy), Oxford (UK) and Santa Fe de Bogota (Colombia), in which the percentage of men with T1D was higher than that in women^2^.

Because the frequency of the HLA-DRB1, -DQB1, and INS-VNTR alleles, as well of the PTPN22 variants, was similar between genders (data not shown), the higher frequency of T1D women in our cohort may be due to other polymorphisms, such as the T allele in the *CD226* rs763361 variant that was suggested by Mattana *et al*.^5^ in the Brazilian cohort, or to environmental factors.

The age at diagnosis of the T1D group was similar to that of Caucasians. The low frequency of pancreatic autoantibodies probably stems from the long duration of diabetes.

The HLA-DR and -DQ alleles and haplotypes associated with T1D susceptibility and protection had similarities to those from European and African-American patients. Aside from the high-risk alleles, including HLA-DR*0301, -*0401, -*0402, and -*0405 and DQB1 *0201 and -*302, which are usually present in both populations, the patients in our study also carried the -*09 allele, which is common in Asian and African populations but neutral in Europeans^16^,^17^. Despite its low frequency (3.7% in patients and 1.4% in controls), the *09 allele conferred a T1D relative risk of 2.67, thus reinforcing its importance as a risk allele for T1D. The HLA-DRB1 *0403, -*0411, -*01, and -*12 were neutral alleles, whereas the DRB1 -0302*, -*07, -*10, -*11, - *13, -*14, and -*15 and DQB1*0402, -*0503, -*0602 and -*0603 alleles conferred protection. The protection conferred by HLA-DRB1*07 was also observed in some, but not all studies conducted in Brazil, perhaps due to insufficient sample size or regional genetic differences^18^.

There was a trend toward protective association with T1D by the DQB1*0301 allele (OR = 0.72; p = 0.003, non-significant). In a meta-analysis, the same allele was causal in Sweden and United Kingdom populations, but protective in Finland, Hungary and Italy^16^. The –DRB1*08 allele also showed a protection trend (OR = 0.58; P = 0.012)^16^. This result is possibly due to its low prevalence in the population from our study (3.4% in T1D patients and 5.7% in controls).

The greatest contributor to T1D was the HLA-DR3/DR4 genotype (OR = 16.5), which was carried by 23.9% of the patients, followed by -DR3/DR3 (OR = 8.9) at 8.7%, -DR4/DR4 (OR = 4.7) at 6.0% and -DR3/DR9 (OR = 4.9) at 2.6%. The frequency of the high-risk DR3/DR4 genotype was intermediary between European (up to 40%)^16^,^17^ and African-American patients (12%).

Despite carrying two risk alleles, the DR4/DR9 genotype did not conferred susceptibility, probably due to the small sample size, which consisted of only 12 patients and five controls. The absence of the HLA -DR3, -DR4, and -DR9 alleles demonstrated lower risk to T1D (OR = 0.1) and occurred in 58.7% of the control population and only 12.5% of patients.

The DRB1*0301-DQB1*0201, -DRB1*0401-DQB1*0302, -DRB1*0402-DQB1*0302, and -DRB1*0405-DQB1*0302 haplotypes conferred T1D predisposition, although the ORs from the DRB1*0401 and *0405 haplotypes containing DQB1*0302 were lower than those reported in European and African-American studies^16^. The DRB1*09-DQB1*0202 haplotype appears to be neutral in Europeans but to confer susceptibility in African Americans, as it was in our analysis The predisposing –DRB1*0404-DQB1*0302 haplotype in Europeans (moderate) and Africans was not confirmed in our patients (OR = 2.05; p = 0.002, not significant). By contrast, the DRB1*07-DQB1*0201 haplotype was protective in our population and in Europeans despite conferring susceptibility in Africans, whereas the DRB1*07-DQB1*0303 haplotype was previously reported to be protective only in Europeans^17^.

The protective effects of the –DRB1*11-DQB1*0301, DRB113*-DQB1*0603, DRB1*14-DQB1*0503 and DRB1*15-DQB1*0602 haplotypes were present in several ethnic groups. By contrast, -DRB1*10-DQB1*0501 was protective only in the cohort from our study, whereas -DRB1*07-DQB1*0202 and –DRB1*11-DQB1*0602 were protective in the cohort from our study and in African Americans^17^, and -DRB1*0401-DQB1*0301 was protective only in Europeans. The haplotypes whose effects differed greatly among European, African-American, and Brazilian cohorts had usually low frequencies (most less than 3%), thus underscoring probable differences in the genetic backgrounds.

Similar data were observed for the non-HLA-loci. The *INS VNTR* I/I genotype was more prevalent in T1D patients (60.7%) than in the control population (32.2%), yielding a relative risk of 3.2 for T1D. A study conducted in São Paulo by Hauache *et al*.^18^ with fewer patients noted a greater frequency of class I alleles, comprising 83.1% of diabetics and 69.3% of controls (OR = 1.98), which is closer to the results observed in Caucasian populations. The frequency of these alleles seems to differ between racial groups. Undlien *et al*.^19^ demonstrated that class I alleles confer susceptibility to T1D in the Caucasian population, but not in black and Japanese populations.

The CT + TT genotypes in *PTPN22 1858C/T* were more prevalent in T1D patients (19%) than in controls (10.6%; p < 0.0001), conferring susceptibility to the disease (OR = 1.97). This risk was significant only in self-reported white individuals, possibly because the T allele is very rare in African-American and Asian populations^20^. In accordance with this result, risk genotypes were present in 26.8% to 42.1% of Caucasian patients (from the USA and Finland) and 16.5% to 25.3% of controls^20^.

Some degree of deviation from Hardy-Weinberg equilibrium is expected in structured populations. For this reason, Hardy-Weinberg deviation analyses were performed for all alleles (HLA) and candidate polymorphisms from the entire sample. One variant of the CTLA4 gene (+49A/G) was not in Hardy-Weinberg equilibrium for the controls and was excluded from further analysis. The literature reports a predisposition in patients from Italy, the UK, and the US who carry the T allele but not in patients from Germany^21^. The T allele was present in 54.5% of patients.

In this study, we confirmed that our population is, in fact, the result of three major ancestral roots: Amerindian, European, and African. This result most likely reflects major milestones in Brazilian history, specifically the European colonization of Indigenous and African slaves. However, the majority of the patients shared a European genetic background, as previously described in a study that included various regions of the country^6^. In fact, European ancestry prevailed in both patients with T1D and the controls, followed by African and Amerindian ancestry.

The predisposition to T1D conferred by HLA-DR/DQ, *PTPN22,* and *INS-VNTR* was also confirmed in our study. The great ethnic diversity resulted in genetic determinants with intermediate frequencies between those of Caucasians and Africans, contributing to risks and protection that were partially discordant from these groups and were likely related to the low/intermediary incidence of T1D in Brazil (8/100,000 per year)^22^.

The correction for population stratification increased the statistical power of our analysis and strengthened the results. Our results highlighted the association of HLA- DRB1*16 and haplotype –DRB1* 07-DQB1*0201 with protection and set DQB1*0501 as neutral.

Our research has a number of limitations. The majority of the population in this study lives in São Paulo. Although these data cannot be extrapolated to our country, it is very representative of Brazil because São Paulo is a cosmopolitan city to which people have converged from all regions of the country as well as from Europe and Asia, with immigrants in the last century. The groups were not homogeneous regarding the mean age, skin color, gender (as stated previously) and the low BMI, which is probably related to the inadequate metabolic control of the patients. The high-resolution genotyping was performed only for the high-risk DR3 and DR4 alleles, which are fundamental to identifying individuals who may benefit from monitoring and preventive treatments and encompasses most of our population. The subtyping of -DQA1 and other neutral or protective DR alleles is important for identifying alleles that provide very strong protection against T1D and individuals who will not progress to the disease. These result are missing in our study, as well the influence of unknown environmental factors. Not all patients were positive for pancreatic autoantibody, possibly due to the cross-sectional nature of the study (implying that not all of them underwent autoantibody determinations at diagnosis) and to the fact that a small percentage of T1D patients are autoantibody negative at the time of diagnosis^2^.

As the frequencies of the alleles referring to the 3 ethnic groups were a continuous, without cut-off point due to the great admixture of our population, it was not possible to define the ethnic-specific HLA alleles and haplotypes associated with T1D.

Predisposition to T1D conferred by HLA-DR/DQ, *PTPN22,* and *INS-VNTR* loci was confirmed in our study. The great ethnic diversity of the population in our study, with contributions from Europeans, Africans and Amerindians, resulted in genetic determinants for T1D with intermediate frequencies between those of Caucasians and Africans, which contributes to partial discordant risk and protection from these groups.

The correction by ancestry indicated that the DRB1*16 allele and -DRB107-DQB1*0201 haplotype were protective against T1D; that the allele DQB1 *0501, which was initially found to be protective, was actually neutral; and that the DRB1*10-DQB1*0501 haplotype was protective. The correction also confirmed that the –DRB1*09-DQB1*0202 haplotype caused susceptibility and that the –DRB1*0302-DQB1*0402, -DRB1*10-DQB1*0501, -DRB1*11-DQB1*0602 and –DRB1*13-DQB1*603 haplotypes were protective in the population, which is similar to the effects observed in African Americans, but not in Caucasians.

## Methods

### Casuistic

The cohort comprised 915 patients with T1D, aged 24.6 ± 13.0 years, and 789 volunteers, aged 28.5 ± 11.5 years, without family history of diabetes or any other autoimmune disease and with normal blood glucose and glycated hemoglobin levels. T1D diagnosis was based on the clinical symptoms of diabetes (weight loss, polyuria, polydipsia) or ketoacidosis at diagnosis, low C peptídeo levels (below the positive cutoff values), and the immediate need of permanent insulin therapy, according to ADA criteria^23^. More recently, the presence of at least one islet autoantibody. Autoantibody determinations started a few years ago. For this reason, many patients underwent this analysis after elapsing many years of diagnosis, explaining those autoantibody negative patients. None was obese nor had a relative with type 2 diabetes or maturity onset of diabetes of the youth (MODY).

The vast majority of patients were attended at the Clinical Hospital. A low percentage was referred by endocrinologists participants in the study. The medical records of patients always included the age at diagnosis and their clinical features. Patients who had any suspicion of non autoimmune diabetes were not included in the cohort.

Most of the cohort lived in São Paulo city. This study was conducted in accordance with ethical principles and following the guidelines contained in the Helsinki Declaration. Approval by the Ethics Committee of the Hospital das Clínicas da Faculdade de Medicina da Universidade de São Paulo and informed consent was obtained from all subjects, parents or guardians were obtained before the research procedures were initiated.

### Glucose, glycated hemoglobin, and C-peptide levels

Fasting plasma glucose was determined using an enzymatic colorimetric assay (LABTEST GOD-ANA, SP, Brazil) and glycated hemoglobin (HbA1c) using high performance liquid chromatography (CLAE; normal values 4.1–6%). Fasting serum C-peptide levels were determined by radioimmunoassay (HCP-20K, Millipore Corporation, Billerica, MA, USA; normal values >0.5 ng/mL; intra- and inter-assay coefficients of variation (CV) were 4.5% and 9.3%, respectively).

### Autoantibodies

Serum levels of the autoantibodies against glutamic acid decarboxylase (GAD65A) and tyrosine phosphatase (IA-2A) were determined by radioimmunoassay (RSR limited, UK; CV < 7%). The normal values for 700 healthy controls (considered 3 standard deviations, SD) were <1.0 IU/mL and <0.8 IU/mL for GAD65A and IA2A, respectively. The sensitivity of both assays was 0.2 IU/mL.

Serum levels of the autoantibodies against Zinc transporter 8 (ZnT8A) were measured by ELISA (KR770-96; Kronus, USA; CV < 7%). This assay detects and quantifies autoantibodies specific to residues R325 and W325 or to non-specific variants of residue 325. The normal value of ZnT8A in 321 healthy controls was defined as ≤16 u/mL (considered 3 SD).

### Molecular study

Genomic DNA was isolated from fresh peripheral blood cells using a conventional salting out method^24^. The HLA-DRB1 and -DQB1 alleles were genotyped using the Micro SSP^TM^ Allele Generic and Specific HLA class II DNA LabType SSO typing system (One-Lambda, INC., USA). Genotypes of the class I and III alleles for *INS-VNTR,* the *CTLA-4*+ 49A/G variant in exon 1, and the PTPN22 C1858T allele were determined by polymerase chain reaction-restriction fragment length polymorphism (PCR-RFLP) using tHph I, BbV and Xcm l^21^,^25^,^26^, respectively.

### Genotyping of ancestry informative markers (AIMs)

A 93 single nucleotide polymorphism (SNP) panel of ancestry autosomal markers (Supplement 1) was used to infer ancestral composition, as described by Nassir *et al*.^7^. These SNPs were obtained from 210 people from the following four populations studied in the first phase of the International HapMap Project, i.e., Nigeria (YRI), European CEPH (CEU), China (CHB) and Japan (JPT)^27^, and were combined with samples from 1,043 people from 51 populations studied in the Human Genome Diversity Panel (HGDP)^28^. All of the samples are freely available online at the website for the projects^28^. These SNPs provide information on the composition of the admixed populations, including sub-Saharan African, European, and Amerindian, and provide results from structured association tests in the context of mixed population groups^7^.

Genotyping of the ancestry informative SNPs was performed using the BeadXpress platform (Illumina, USA). A total of 1,284 samples (632 patients with diabetes and 652 controls) were analyzed using the Goldengate genotyping assay (Veracode, Illumina, USA) according to manufacturer’s procedures. The data were examined using the Genome Studio software^29^.

### Quality control of samples and genotyped SNPs

To reduce the occurrence of false associations, quality control filtering of the samples and SNPs was performed by considering the genotyping efficiency (call rate >95%), gender concordance and SNP performance (SNP gene train score usually >0.90) (15). Of the 93 AIMs, four, rs11652805, rs4717865, rs6548616, and rs1950993, were excluded from the analysis owing to low performance genotyping, which resulted in 89 SNPs that were effectively analyzed.

Deviations from the Hardy-Weinberg equilibrium were used as a quality control filter for the SNPs, and the 89 SNPs were all in equilibrium (>0.05).

## Additional Information

**How to cite this article**: Gomes, K. F. B. *et al*. The influence of population stratification in genetic markers associated with type 1 diabetes. *Sci. Rep.* **7**, 43513; doi: 10.1038/srep43513 (2017).

**Publisher's note:** Springer Nature remains neutral with regard to jurisdictional claims in published maps and institutional affiliations.

## Supplementary Material

Supplementary Information

## Figures and Tables

**Table 1:** Demographic data of patients with type 1 diabetes and health controls.

	T1D patients n(%)915	Mean ± SD	Controls n(%) 789	Mean ± SD	P^a^
Age (years)		24.6 ± 13.0		28.5 ± 11.5	<0.0001
Disease duration (years)		12.4 ± 10.6			
Age at diagnosis (years)		12.3 ± 8.4			
Gender					
Male	388 (42.4%)		477 (60.5%)		<0.0001^a^
Female	527 (57.6%)		312 (39.5%)		
BMI (kg/m^2^)	21.8 ± 4.3		24.2 ± 3.5		<0.0001^b^
Self-reported skin Color					
White	729 (81.7%)		490 (65.6%)		<0.0001^a^
Brown	137 (15.4%)		208 (27.8%)		<0.0001^a^
Black	21 (2.4%)		44 (5.9%)		0.0003^a^
Yellow	5 (0.6%)		5 (0.7%)		0.7782^a^
Glycemia (mg/dL)	189.3 ± 113.7		83.5 ± 9.8		<0.0001
HbA1c (%)	8.4 ± 2.2		5.1 ± 0.4		<0.0001
HbA1c(mmol/mol)	68.3 ± 2.2		32.2 ± 0.4		<0.0001
C-peptide (ng/dL)	0.37 ± 0.31		2.21 ± 1.73		<0.0001

T1D = type 1 diabetes mellitus, n = number of subjects, SD = standard deviation.

^a^Fisher’s exact test.

^b^Student T test, HbA1c = glycated hemoglobin, BMI = body mass index.

**Table 2:** HLA-DRB1 alleles distribution in patients with type 1 diabetes and health controls.

DRB1	T1D	Controls	OR	CI 95%	p	p
alleles	n (%)	n(%)			no-structured	structured
1	119 (8.5%)	158 (10.5%)	0.79	(0.62–1.02)	0.062	0.108
301	400 (28.7%)	121 (8.1%)	4.58	(3.68–5.69)	<0.0001	<0.001
302	1 (0.1%)	20 (1.3%)	0.05	(0.01–0.39)	<0.0001	<0.001
401	127 (9.1%)	52 (3.5%)	2.79	(2.00–3.90)	<0.0001	<0.001
402	98 (7.0%)	27 (1.8%)	4.12	(2.67–6.35)	<0.0001	<0.001
403	7 (0.5%)	11 (0.8%)	0.68	(0.26–1.76)	0.578	—
404	38 (2.7%)	21 (1.4%)	1.97	(1.15–3.37)	0.0119	0.007
405	142 (10.2%)	18 (1.2%)	9.32	(5.68–15.31)	<0.0001	<0.001
407	3 (0.2%)	8 (0.5%)	0.4	(0.1–1.5)	0.1639	0.1345
408	4 (0.3%)	7 (0.5%)	0.61	(0.2–2.1)	0.4310	0.5316
411	3 (0.2%)	9 (0.6%)	0.36	(0.09–1.32)	0.186	0.1070
7	79 (5.7%)	177 (11.8%)	0.45	(0.34–0.59)	<0.0001	<0.001
8	47 (3.4%)	85 (5.7%)	0.58	(0.40–0.83)	0.0030	0.012
9	51 (3.7%)	21 (1.4%)	2.67	(1.59–4.46)	<0.0001	<0.001
10	10 (0.7%)	33 (2.2%)	0.32	(0.16–0.65)	0.0010	0.002
11	53 (3.8%)	159 (10.6%)	0.33	(0.24–0.46)	<0.0001	<0.001
12	13 (0.9%)	24 (1.6%)	0.58	(0.29–1.14)	0.1093	0.168
13	93 (6.7%)	207 (13.8%)	0.45	(0.34–0.58)	<0.0001	<0.001
14	14 (1.0%)	70 (4.7%)	0.21	(0.12–0.37)	<0.0001	<0.001
15	28 (2.0%)	177 (11.8%)	0.15	(0.10–0.23)	<0.0001	<0.001
16	28 (2.0%)	59 (3.9%)	0.5	(0.32–0.79)	0.0024	0.001
Others	45 (3.2%)	51 (3.4%)				

Association with Type 1 diabetes before and after correction for population stratification T1D = type 1 diabetes mellitus; n = number of individuals; OR = odds ratio; CI = confidence interval; *p* no-structured = significance level before STRAT analysis; *p* structured = significance level after STRAT analysis.

Twenty-one alleles with total number in patients plus controls greater than 10 (0.4%) were included. *P* required for statistical significance after Bonferroni correction for multiple tests <0.0023. Rare alleles were included in others.

**Table 3:** HLA-DQB1 alleles distribution in patients with type 1 diabetes and controls.

DQB1	T1D	Controls	OR	CI 95%	p	p
alleles	n (%)	n(%)			no-structured	structured
201	423 (30.8%)	156 (11.2%)	3.52	(2.88–4.31)	<0.0001	<0.001
202	94 (6.9%)	136 (9.8%)	0.68	(0.51–0.89)	0.0051	0.008
301	140 (10.2%)	189 (13.6%)	0.72	(0.57–0.91)	0.0057	0.003
302	371 (27.0%)	139 (10.0%)	3.33	(2.69–4.12)	<0.0001	<0.001
303	27 (2.0%)	32 (2.3%)	0.85	(0.51–1.43)	0.5399	0.739
319	1 (0.1%)	11 (0.8%)	0.091	(0.012–0.708)	0.0041	0.0052
401	5 (0.4%)	6 (0.4%)	0.84	(0.26–2.77)	0.7773	—
402	32 (2.3%)	81 (5.8%)	0.38	(0.25–0.58)	<0.0001	<0.001
501	134 (9.8%)	190 (13.7%)	0.68	(0.54–0.86)	0.0014	0.007
502	30 (2.2%)	51 (3.7%)	0.59	(0.37–0.93)	0.0206	0.01
503	7 (0.5%)	40 (2.9%)	0.17	(0.08–0.39)	<0.0001	<0.001
601	6 (0.4%)	8 (0.6%)	0.76	(0.26–2.19)	0.6071	0.454
602	30 (2.2%)	201 (14.5%)	0.13	(0.09–0.19)	<0.0001	<0.001
603	16 (1.2%)	90 (6.5%)	0.17	(0.09–0.29)	<0.0001	<0.001
604	47 (3.4%)	39 (2.8%)	1.23	(0.79–1.89)	0.3518	0.691
609	4 (0.3%)	12 (0.9%)	0.335	(0.108–1.042)	0.0474	
Others	10 (0.7%)	30 (2.2%)				
Total	1372	1388				

Association with Type 1 diabetes before and after correction for population stratification T1D = type 1 diabetes mellitus; n = number of individuals; OR = odds ratio; CI = confidence interval; *p* no-structured = level of significance before strat analysis; *p* structured = level of significance after strat analysis.

Sixteen alleles, with total number in patients plus controls greater than 10 (0.4%), were included. *P* required for statistical significance after Bonferroni correction for multiple tests <0.003. Rare alleles were included in others.

**Table 4:** HLA-DRB1/DRB1 genotypes distribution in patients with type 1 diabetes mellitus and controls.

Genotypes	T1D n(%)	Controls n(%)	OR	CI 95%	p no-strutured	p structured
DR3/DR3	61 (8.7%)	8 (1.1%)	8.88	(4.22–18.70)	<0.0001	<0.001
DR3/DR4	167 (23.9%)	14 (1.9%)	16.53	(9.48–28.85)	<0.0001	<0.001
DR3/DR9	18 (2.6%)	4 (0.5%)	4.94	(1.66–14.66)	0.0015	0.002
DR3/DRX	110 (15.8%)	120 (16.0%)	0.98	(0.74–1.30)	0.9004	0.802
DR4/DR4	42 (6.0%)	10 (1.3%)	4.74	(2.36–9.52)	<0.0001	<0.001
DR4/DR9	12 (1.7%)	5 (0.7%)	2.61	(0.91–7.44)	0.0632	0.105
DR4/DRX	181 (25.9%)	137 (18.3%)	1.57	(1.22–2.01)	0.0004	0.002
DR9/DR9	1 (0.1%)	0 (0.0%)	—	—	0.2998	—
DR9/DRX	19 (2.7%)	12 (1.6%)	1.72	(0.83–3.57)	0.1405	0.159
DRX/DRX	87 (12.5%)	440 (58.7%)	0.1	(0.078–0.13)	<0.0001	<0.001

Association with Type 1 diabetes before and after correction for population stratification T1D = type 1 diabetes mellitus; n = number of individuals; OR = odds ratio; CI = confidence interval; *p* no-structured = level of significance before strat analysis; *p* structured = level of significance after strat analysis. *P* required for statistical significance after a Bonferroni correction for multiple tests −<0.005.

**Table 5:** Distribution of the HLA -DRB1/DQB1 haplotypes in patients with type 1 diabetes mellitus and normal controls before and after correction for population stratification

Haplotypes	T1D n(%)	Controls n(%)	OR	CI 95%	p no-structured	p strutured
01–0501	114 (8.3%)	142 (10.2%)	0.79	(0.61–1.03)	0.0819	0.068
0301–0201	389 (28.4%)	109 (7.9%)	4.64	(3.69–5.83)	<0.0001	<0.001
0301–0202	7 (0.5%)	4 (0.3%)	1.77	(0.52–6.07)	0.3546	—
0302–0402	0 (0.0%)	17 (1.2%)	0.29	(0.00–0.048)	<0.0001	<0.001
0401–0301	18 (1.3%)	7 (0.5%)	2.62	(1.09–6.29)	0.0251	0.107
0401–0302	105 (7.7%)	40 (2.9%)	2.79	(1.92–4.05)	<0.0001	<0.001
0402–0301	11 (0.8%)	2 (0.1%)	5.6	(1.24–25.32)	0.0116	0.008
0402–0302	87 (6.3%)	23 (1.7%)	4.02	(2.52–6.40)	<0.0001	<0.001
0403–0302	4 (0.3%)	8 (0.6%)	0.5	(0.152–1.679)	0.2555	0.2643
0404–0302	32 (2.3%)	16 (1.2%)	2.05	(1.12–3.75)	0.0178	0.002
0405–0302	110 (8.0%)	17 (1.2%)	7.03	(4.19–11.78)	<0.0001	<0.001
0411–0302	3 (0.2%)	8 (0.6%)	0.38	(0.100–1.428)	0.1359	0.1367
07–0201	16 (1.2%)	38 (2.7%)	0.42	(0.23–0.75)	0.0029	0.001
07–0202	55 (4.0%)	109 (7.9%)	0.49	(0.35–0.68)	<0.0001	<0.001
08–0301	3 (0.2%)	12 (0.9%)	0.25	(0.07–0.89)	0.021	0.067
08–0402	29 (2.1%)	58 (4.2%)	0.49	(0.31–0.78)	0.0019	0.004
09–0202	25 (1.8%)	6 (0.4%)	4.27	(1.75–10.45)	0.0005	<0.001
09–0303	17 (1.2%)	7 (0.5%)	2.47	(1.02–5.99)	0.0376	0.234
10–0501	10 (0.7%)	32 (2.3%)	0.31	(0.15–0.63)	0.0007	<0.001
11–0301	44 (3.2%)	92 (6.6%)	0.47	(0.32–0.67)	<0.0001	<0.001
11–0602	0 (0.0%)	27 (1.9%)	0.02	(0.00–0.30)	<0.0001	<0.001
12–0301	12 (0.9%)	19 (1.4%)	0.64	(0.31–1.31)	0.218	0.457
13–0301	6 (0.4%)	16 (1.2%)	0.38	(0.147–0.965)	0.0346	0.0354
13–0303	3 (0.2%)	16 (1.2%)	0.19	(0.055–0.646)	0.003	0.0041
13–0602	13 (0.9%)	15 (1.1%)	0.88	(0.41–1.85)	0.727	0.582
13–0603	17 (1.2%)	86 (6.2%)	0.19	(0.11–0.32)	<0.0001	<0.001
13–0604	46 (3.4%)	38 (2.7%)	1.23	(0.79–1.91)	0.347	0.749
13–0609	3 (0.2%)	12 (0.9%)	0.25	(0.071–0.892)	0.021	0.0321
14–0301	1 (0.1%)	9 (0.6%)	0.11	(0.01–0.88)	0.0119	—
14–0501	3 (0.2%)	9 (0.6%)	0.34	(0.09–1.24)	0.0862	—
14–0503	7 (0.5%)	38 (2.7%)	0.18	(0.08–0.41)	<0.0001	<0.001
15–0601	4 (0.3%)	7 (0.5%)	0.58	(0.17–1.97)	0.375	—
15–0602	17 (1.2%)	152 (11.0%)	0.1	(0.06–0.16)	<0.0001	<0.001
16–0301	3 (0.2%)	14 (1.0%)	0.21	(0.06–0.75)	0.008	0.005
16–0502	25 (1.8%)	42 (3.0%)	0.59	(0.36–0.98)	0.0399	0.012
Others	116 (8.5%)	189 (13.6%)				
Total	1355	1436				

T1D = type 1 diabetes mellitus; n = number of individuals; OR = odds ratio; CI = confidence interval; *p* no-structured = level of significance before strat analysis; *p* structured = level of significance after strat analysis. Thirty five haplotypes with total number in patients plus controls greater than 10 were included (0.4%). *P* required for statistical significance after Bonferroni correction for multiple tests <0.0013. Rare alleles were included in others.

**Table 6:** Genotypic frequencies of polymorphic variants related to T1D of patients with type 1 diabetes and controls before and after correction for population stratification

Genes	Genotypes	T1D n(%)	Controls n (%)	OR	CI 95%	p no structured	p structured
*INS-VNTR*	I/I	284 (60.7%)	120 (32.2%)	3.25	2.44–4.33	<0.0001	<0.001
	I/III + III/III	184 (39.3%)	253 (67.8%)				
*PTPN22*	cc	375 (81%)	614 (89.4%)				
(1858T)	ct + tt	88 (19.0%)	73 (10.6%)	1.97	1.40–2.75	<0.0001	<0.001
*CTLA4*	aa	296 (45.5%)	276 (45.0%)				
(+49 A/G)	ag + gg	355 (54.5%)	338 (55.0%)	—	—	—	—

T1D = type 1 diabetes mellitus; n = number of individuals; OR = odds ratio; CI = confidence interval; *INS-VNTR* = variation in the number of repetitions of nucleotides 5′ of the insulin gene; *PTPN22* = tyrosine-protein phosphatase non-receptor type 22; *CTLA4* = Cytotoxic T lymphocyte antigen 4.
