# Supplementary material for: The influence of population stratification on genetic markers associated with type 1 diabetes
Source: Sci Rep. 2017 Mar 6;7:43513. doi: 10.1038/srep43513 (PMC5338024; doi:10.1038/srep43513)
Supplement: Supplementary Information [file srep43513-s1.docx]

The influence of population stratification on genetic markers associated with type 1 diabetes

Karla Fabiana Brasil Gomes, Aritânia Sousa Santos, Cintia Semzezem, Márcia Regina Correia, Luciano Abreu Brito, Marcelo Ortega Ruiz, Rosa Tsuneshiro Fukui, Sergio Russo Matioli, Maria Rita Passos-Bueno & Maria Elizabeth Rossi da Silva

Supplement - *SNP Ancestry Informative Markers.*

| **NCBI** | **SNP_Assay** | **Strand** | **Alelle 1** | **Alelle 2** | | **Chr** | | **Assembly** | | **Sequence** | |  |
| --- | --- | --- | --- | --- | --- | --- | --- | --- | --- | --- | --- | --- |
| rs10108270 | C__30263561_10 | Forward | A | C | | 8 | | 36 | | 4178201 | |  |
| rs10236187 | C____328256_10 | Forward | A | C | | 7 | | 36 | | 139093846 | |  |
| rs1040045 | C___8767011_10 | Forward | A | G | | 6 | | 36 | | 4692158 | |  |
| rs1040404 | C___2985471_10 | Reverse | A | G | | 1 | | 36 | | 166426514 | |  |
| rs10496971 | C__30021395_20 | Forward | G | T | | 2 | | 36 | | 145486413 | |  |
| rs10510228 | C___9471400_10 | Forward | A | G | | 3 | | 36 | | 2183832 | |  |
| rs10512572 | C__30205773_20 | Forward | A | G | | 17 | | 36 | | 67023694 | |  |
| rs10513300 | C__30087237_20 | Reverse | C | T | | 9 | | 36 | | 119170027 | |  |
| rs10839880 | C__26880884_10 | Forward | C | T | | 11 | | 36 | | 7806892 | |  |
| rs11227699 | C__27162924_10 | Forward | A | G | | 11 | | 36 | | 66655068 | |  |
| rs11652805 | C__31084340_10 | Reverse | C | T | | 17 | | 36 | | 60417613 | |  |
| rs12130799 | C__32221116_10 | Forward | A | G | | 1 | | 36 | | 55435960 | |  |
| rs12439433 | C___1447138_10 | Reverse | A | G | | 15 | | 36 | | 34007327 | |  |
| rs12544346 | C___8237454_10 | Forward | A | G | | 8 | | 36 | | 86611868 | |  |
| rs12629908 | C___1508579_10 | Reverse | A | G | | 3 | | 36 | | 122005406 | |  |
| rs12657828 | C___3164411_10 | Reverse | A | G | | 5 | | 36 | | 79121482 | |  |
| rs1296819 | C___2618285_20 | Forward | A | C | | 22 | | 36 | | 16456546 | |  |
| rs1325502 | C___9030949_10 | Forward | A | G | | 1 | | 36 | | 42132857 | |  |
| rs13400937 | C__32187474_20 | Forward | G | T | | 2 | | 36 | | 79718431 | |  |
| rs1369093 | C___7511789_10 | Reverse | C | T | | 4 | | 36 | | 73464055 | |  |
| rs1407434 | C___7550746_10 | Forward | A | G | | 1 | | 36 | | 184415655 | |  |
| rs1471939 | C___8793707_20 | Reverse | C | T | | 8 | | 36 | | 28997224 | |  |
| rs1513181 | C___1519732_10 | Reverse | A | G | | 3 | | 36 | | 190057690 | |  |
| rs1760921 | C___8833162_10 | Reverse | C | T | | 14 | | 36 | | 19887971 | |  |
| rs1837606 | C___2910912_10 | Reverse | C | T | | 11 | | 36 | | 15794713 | |  |
| rs1871428 | C___1903207_10 | Reverse | A | G | | 6 | | 36 | | 168408609 | |  |
| rs1950993 | C___2789623_10 | Forward | G | T | | 14 | | 36 | | 57308440 | |  |
| rs200354 | C___2968151_20 | Forward | G | T | | 14 | | 36 | | 98445074 | |  |
| rs2030763 | C____443622_10 | Reverse | A | G | | 3 | | 36 | | 181447421 | |  |
| rs2073821 | C__16163355_10 | Forward | C | T | | 9 | | 36 | | 134922943 | |  |
| rs2125345 | C__15885530_10 | Reverse | C | T | | 17 | | 36 | | 71293786 | |  |
| rs214678 | C___2403618_10 | Reverse | C | T | | 12 | | 36 | | 45963217 | |  |
| rs2306040 | C___3069822_20 | Reverse | C | T | | 9 | | 36 | | 92681020 | |  |
| rs2330442 | C__11837185_10 | Forward | A | G | | 7 | | 36 | | 42346596 | |  |
| rs2357442 | C__16209136_20 | Reverse | A | C | | 14 | | 36 | | 51677717 | |  |
| **NCBI** | **SNP_Assay** | **Strand** | **Alelle 1** | | **Alelle 2** | | **Chr** | | **Assembly** | | **Sequence** | |
| rs6541030 | C__16226232_10 | Reverse | C | | T | | 6 | | 36 | | 51719429 | |
| rs2416791 | C__16234767_10 | Forward | A | | G | | 12 | | 36 | | 11592755 | |
| rs2504853 | C__16252570_10 | Reverse | C | | T | | 6 | | 36 | | 12643097 | |
| rs260690 | C____790944_10 | Forward | A | | C | | 2 | | 36 | | 108946170 | |
| rs2627037 | C__15900715_10 | Forward | A | | G | | 2 | | 36 | | 179314783 | |
| rs2702414 | C__27311585_10 | Reverse | A | | G | | 4 | | 36 | | 179636517 | |
| rs2946788 | C____302128_10 | Reverse | G | | T | | 11 | | 36 | | 23967106 | |
| rs2986742 | C__16188366_10 | Forward | C | | T | | 1 | | 36 | | 6472963 | |
| rs3118378 | C___7770343_10 | Reverse | A | | G | | 1 | | 36 | | 68622275 | |
| rs316598 | C___2949512_10 | Reverse | C | | T | | 5 | | 36 | | 2417626 | |
| rs316873 | C____722586_10 | Reverse | C | | T | | 1 | | 36 | | 240409127 | |
| rs32314 | C___2270718_10 | Reverse | C | | T | | 7 | | 36 | | 32145649 | |
| rs3737576 | C__27471358_10 | Reverse | C | | T | | 1 | | 36 | | 101482151 | |
| rs3745099 | C__27472381_10 | Reverse | A | | G | | 19 | | 36 | | 57593717 | |
| rs3784230 | C___2770233_10 | Forward | A | | G | | 14 | | 36 | | 104750100 | |
| rs385194 | C___1121830_10 | Reverse | A | | G | | 4 | | 36 | | 85528102 | |
| rs3907047 | C___8948366_10 | Forward | C | | T | | 20 | | 36 | | 53434321 | |
| rs3943253 | C__11860358_10 | Reverse | A | | G | | 8 | | 36 | | 13403871 | |
| rs4463276 | C____493379_10 | Forward | A | | G | | 6 | | 36 | | 145097024 | |
| rs4666200 | C__27891561_10 | Forward | A | | G | | 2 | | 36 | | 29391915 | |
| rs4670767 | C__27978607_10 | Forward | G | | T | | 2 | | 36 | | 37794900 | |
| rs4717865 | C__11448835_10 | Reverse | A | | G | | 7 | | 36 | | 73092135 | |
| rs4746136 | C__27913671_10 | Forward | A | | G | | 10 | | 36 | | 74971000 | |
| rs4781011 | C__11293224_10 | Forward | G | | T | | 16 | | 36 | | 10882812 | |
| rs4821004 | C___2465604_1_ | Forward | C | | T | | 22 | | 36 | | 30696359 | |
| rs4908343 | C___2494120_10 | Reverse | A | | G | | 1 | | 36 | | 27804285 | |
| rs4918842 | C___3001048_10 | Reverse | C | | T | | 10 | | 36 | | 115306802 | |
| rs4984913 | C__27950884_10 | Forward | A | | G | | 16 | | 36 | | 680467 | |
| rs5768007 | C__29548230_10 | Reverse | C | | T | | 22 | | 36 | | 46586536 | |
| rs6422347 | C__29040411_10 | Forward | C | | T | | 5 | | 36 | | 177795689 | |
| rs6451722 | C___2938090_10 | Forward | A | | G | | 5 | | 36 | | 43747135 | |
| rs4951629 | C___2662380_10 | Reverse | C | | T | | 7 | | 36 | | 151504786 | |
| rs647325 | C____778913_10 | Reverse | A | | G | | 1 | | 36 | | 18043473 | |
| rs6548616 | C__29071253_10 | Reverse | C | | T | | 3 | | 36 | | 79482265 | |
| rs705308 | C___2637064_10 | Reverse | A | | C | | 7 | | 36 | | 97533299 | |
| rs7238445 | C__28994653_10 | Forward | A | | G | | 18 | | 36 | | 48035542 | |
| rs731257 | C_____14517_10 | Forward | A | | G | | 7 | | 36 | | 12635776 | |
| rs734873 | C____788020_10 | Forward | A | | G | | 3 | | 36 | | 149233045 | |
| rs7421394 | C__31183706_10 | Reverse | A | | G | | 2 | | 36 | | 14673800 | |
| rs7554936 | C__26139689_10 | Reverse | C | | T | | 1 | | 36 | | 149389113 | |
| rs7657799 | C__29422763_10 | Forward | G | | T | | 4 | | 36 | | 105594872 | |
| rs772262 | C___8340116_10 | Forward | A | | G | | 12 | | 36 | | 54450001 | |

continua

| **NCBI** | | **SNP_Assay** | | **Strand** | **Alelle 1** | **Alelle 2** | **Chr** | **Assembly** | **Sequence** | |
| --- | --- | --- | --- | --- | --- | --- | --- | --- | --- | --- |
| rs7745461 | C__29245583_10 | | Forward | | A | G | 6 | 36 | 22019595 |  |
| rs7803075 | C___2130393_10 | | Reverse | | A | G | 7 | 36 | 130392606 |  |
| rs7844723 | C___1552011_10 | | Reverse | | C | T | 8 | 36 | 122977684 |  |
| rs798443 | C___8914321_10 | | Forward | | A | G | 2 | 36 | 7885726 |  |
| rs7997709 | C__30127919_10 | | Reverse | | C | T | 13 | 36 | 33745737 |  |
| rs8035124 | C____486094_10 | | Reverse | | A | C | 15 | 36 | 89906712 |  |
| rs8113143 | C___2882928_10 | | Reverse | | A | C | 19 | 36 | 38344087 |  |
| rs818386 | C___1395556_10 | | Reverse | | C | T | 16 | 36 | 63964209 |  |
| rs870347 | C___3052139_10 | | Reverse | | A | C | 5 | 36 | 6898035 |  |
| rs874299 | C___9605053_10 | | Reverse | | C | T | 18 | 36 | 73185272 |  |
| rs9319336 | C__27328815_10 | | Forward | | C | T | 13 | 36 | 26522356 |  |
| rs948028 | C___8799834_10 | | Reverse | | A | C | 11 | 36 | 120149657 | |
| rs9522149 | C__30502208_20 | | Reverse | | C | T | 13 | 36 | 110625168 | |
| rs9530435 | C__27192660_10 | | Reverse | | C | T | 13 | 36 | 74891888 | |
| rs9809104 | C__30049893_10 | | Reverse | | C | T | 3 | 36 | 39121433 | |
| rs9845457 | C___1478361_10 | | Reverse | | A | G | 3 | 36 | 137397166 | |
